# Microscopy assessment of a fluorescence [^18^F] flortaucipir analog (T726) shows neuropathological overlap with 3R and 4R tau lesions

**DOI:** 10.1002/alz.14330

**Published:** 2024-10-22

**Authors:** Rodolfo G. Gatto, Youssef Hossam, R. Ross Reichard, Val J. Lowe, Jennifer L. Whitwell, Keith A. Josephs

**Affiliations:** ^1^ Department of Neurology Mayo Clinic Rochester Minnesota USA; ^2^ Department of Laboratory Medicine and Pathology Mayo Clinic Rochester Minnesota USA; ^3^ Department of Radiology Mayo Clinic Rochester Minnesota USA

**Keywords:** flortaucipir, frontotemporal lobar degeneration, Pick's disease, progressive supranuclear palsy, T726, tauopathy

## Abstract

**BACKGROUND:**

[^18^F] flortaucipir (FTP) binding to paired helical filament (PHF) tau in Alzheimer's disease (AD) is well accepted. Binding to 3R and 4R tau in frontotemporal lobar degeneration (FTLD) is controversial. We aimed to investigate whether an FTP fluorescent analog (T726) can help shed light on this controversy.

**METHOD:**

We assessed T726 binding to amyloid beta (Aβ) and different tau isoforms in nine subjects (one control, three with Alzheimer's disease [AD], and five with FTLD) with different 3R and 4R tauopathies using fluorescence confocal microscopy.

**RESULTS:**

T726 did not colocalize with Aβ but showed significant co‐localization with PHF tau in AD. We also observed some, albeit limited, co‐localization of T726 with 3R and 4R tau lesions in FTLD.

**DISCUSSION:**

This study's findings support FTP binding to some 3R and 4R tau lesions in FTLD. Further studies are needed to understand the biology of why FTP binds some but not all FTLD tau lesions.

**Highlights:**

- Flortaucipir analog (T726) showed significant co‐localization with paired helical filament (PHF) tau in Alzheimer's disease (AD).
- Colocalization between T726 with 3R and 4R tau lesions was observed in frontotemporal lobar degeneration (FTLD).
- Not all 4R tau lesions bind to T726 across different FTLD brain regions.

## Graphical abstract

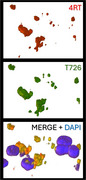

## 1 BACKGROUND

The growing incidence of frontotemporal lobar degeneration (FTLD) constitutes a global concern due to the increase in human life expectancy and the prevalence of this disease across a relatively active young segment of the population.^1^ Neurodegeneration in FTLD is related to at least three classes of proteins: the microtubule‐associated protein tau, the trans‐activation response element (TAR) DNAbinding protein of 43 kDa, and the fused in Sarcoma, Ewing sarcoma breakpoint region 1, and TATA‐box binding protein associated factor 15 (FET) protein family.^2^ Therefore, it is essential to detect the specific underlying protein in FTLD. Recently, positron emission tomography (PET) tracers that can detect tau have become available. Of these tau tracers, the most commonly utilized in patients with cognitive impairment is [^18^F] flortaucipir (FTP).^3^, ^4^, ^5^ FTP was developed initially to detect tau pathology in cognitive impairment due to Alzheimer's disease (AD).^6^, ^7^ However, FTP has also been applied to FTLD, including cases with underlying tauopathies.^8^, ^9^, ^10^, ^11^, ^12^

A handful of studies to date have shown that FTP uptake strongly correlates with underlying tau pathology in AD, which is characterized by pair helical filament (PHF) tau with a mixture of three repeat tau (3RT) and four repeat tau (4RT).^13^, ^14^, ^15^, ^16^, ^17^ Hence, FTP is well‐established as a biomarker of AD tau neuropathology.^18^, ^19^, ^20^, ^21^ In addition to AD, however, some research centers have also applied FTP PET to patients with FTLD, including those with 4R‐specific tauopathy and those with 3R‐specific tauopathy (i.e., those with FTLD‐tau).^9^, ^10^, ^11^, ^12^, ^22^ Many of these PET studies have found increased FTP uptake in specific regions in FTLD‐tau compared to healthy controls.^23^ However, researchers have come to different conclusions regarding the increased FTP uptake in FTLD.^24^ Some researchers have concluded that uptake in FTLD‐tau is likely nonspecific and is all due to off‐target binding, that is, uptake is unrelated to the underlying 4R or 3R tau lesions in FTLD.^25^, ^26^ On the other hand, some researchers have supported that FTP uptake in FTLD‐tau could be due to the ligand directly binding to 4R or 3R tau lesions.^27^

Determining whether FTP uptake in FTLD‐tau is all due to off‐target binding or whether FTP does bind to some 4R and 3R tau in FTLD‐tau is difficult, given the lack of ability to directly assess PET ligand binding. However, an FTP fluorescent carbazole analog (T726) is available to help address this question. T726 has been studied in a few AD cases, whereby it has been shown to bind to premature tau aggregates^28^ preferentially. T726 has not been assessed in cases with 3RT or 4RT. In this postmortem study, we utilize this FTP analog to determine whether it can help shed light on whether FTP binds to 4RT and 3RT lesions in FTLD‐tau diseases.

## 2 MATERIALS AND METHODS

### 2.1 Participants

Brain samples were acquired from the Mayo Clinic neuropathological database in Rochester, Minnesota, for all patients who had been enrolled in a National Institutes of Health (NIH)–funded study by the Neurodegenerative Research Group (NRG; principal investigators [P.I.s]: K.J. and J.W.) and had died with brain autopsy examination performed at Mayo Clinic, Minnesota, between January 1, 2017, and July 31, 2022. We identified a total of nine patients. Of these nine patients, three were pathologically diagnosed as having AD neuropathological changes (ADNC): One had evidence of low ADNC (L‐AD), one had intermediate ADNC (I‐AD), and the third had high ADNC (H‐AD).^29^, ^30^, ^31^, ^32^ Five patients had FTLD‐tau, including two with progressive supranuclear palsy (PSP‐1 and PSP‐2) and one each with corticobasal degeneration (CBD), glial globular tauopathy (GGT) type 1, and Pick's disease (PiD). PSP, CBD, and GGT are all 4R tauopathies, whereas PiD is a 3R tauopathy. We also included a subject, as a control, who died without any evidence of ADNC or FTLD neuropathology.

**RESEARCH IN CONTEXT**

1. **Systematic review**: Experimental and clinical data support the notion that [^18^F] flortaucipir binds primarily to paired helical filament (PHF; 3R+4R) tau in Alzheimer´s disease (AD). Little is known about the binding of [^18^F] flortaucipir to other tau isoforms (4R and 3R) that characterize some cases of frontotemporal lobar degeneration (FTLD).
2. **Interpretation**: Immunofluorescence confocal microscopy utilizing a flortaucipir analog, T726, supports the binding of flortaucipir to PHF‐1 in AD, as well as some binding to 3R and 4R tau in FTLD.
3. **Future directions**: To determine the structural and molecular characteristics of those 4R tau lesions that bind to flortaucipir compared to 4R tau lesions that do not bind.

The Mayo Clinic Institutional Review Board (IRB) approved the study, and all patients or proxies consented to the research study. (The proxies provided consent for patients when needed.) The study followed the ethical standards of the Committee on Human Experimentation at Mayo Clinic by the Helsinki Declaration of 1975.

### 2.2 Quantitative neuropathology

All patients had a standardized neuropathologic evaluation following accepted published methodologies.^29^, ^33^ Diagnoses were made by one neuropathologist (R.R.R.). The degree of ADNC was determined using standard methods.^34^ Tau detection was performed using anti‐AT8 antibodies (Thermo Fisher Scientific, MN1020, 1:100). This antibody is used because it binds to all tau species.^35^, ^36^ For each patient, we assessed the burden of tau on AT8 stained slides in four specific brain regions: hippocampus (HC), inferior temporal cortex (Temp‐ccx), superior middle frontal cortex (SMFG), and basal ganglia (BG). Additional immunostainings were performed with an anti‐three repeat tau isoform (RD3, clone 8E6/C11, Millipore, Cat. # 05‐803, Lot # 2153147, 1:100)^37^ in the amygdala and cortical temporal regions of the PiD case. For imaging, we used a regular scanning microscope (Grundium Ocus 40). Quantitative determination of tau burden was completed using previously described thresholding and masking methods.^38^, [Fig alz14330-fig-0001]

### 2.3 Fluorescent immunohistochemical and confocal imaging methods

Following general deparaffinization of the samples as described, the specimen samples were incubated with TrueBlack^®^ lipofuscin autofluorescence quencher (Biotium, #23007) and with T726 (the fluorescence analog of FTP), 1:100 for 2 h (Xia et al.). Samples were then stained with four different antibodies: (1) beta‐Amyloid (Fisher, BAM01; 6F/3D, MA5‐11617, 1:50) to detect amyloid beta (Aβ) in senile plaques^39^, ^40^; (2) Ab39 anti‐tau (Abcam, GT‐38, ab246808, 1:400), an antibody that selectively binds to an AD specific conformation of pathological tau^41^, ^42^; (3) anti‐tau PHF‐1 (Abcam, ab184951, mouse monoclonal 1:50) that detects a range of PHF tau^43^; (4) anti 4R tau (RD4, clone 1E1/A6, Millipore, 05‐804, 1:50),^37^ and (5) RD3 (clone 8E6/C11, Millipore, Cat. # 05‐803, Lot # 2153147, 1:200). A secondary antibody, goat anti‐mouse Alexa 594, 1:200, was applied after incubation with each primary. Slides were mounted VECTASHIELD DAPI for counterstaining. Imaging was performed by confocal microscopy (LSM 780, Zeiss) and analysis on ImageJ. For fluorescence studies, we used tissue from the SMFG, the hippocampus, the temporal gyrus, and the BG regions. DAPI, 4′,6‐diamidino‐2‐phenylindole nuclear counterstaining.[Fig alz14330-fig-0002]

### 2.4 Statistical methods

Spatial co‐localization analyses were performed applying the Pearson's co‐localization coefficient (PCC) obtained from ImageJ, and standard methods described extensively in the literature.^44^, ^45^, ^46^

## 3 RESULTS

### 3.1 Case characterization

The demographic and neuropathologic features of this cohort have been published previously.^38^ The age at death of the nine patients ranged from 68 to 87 years of age and included five men and four women. The ages of the AD patients ranged from 78 to 80 years, with no apparent differences between time to scan to death (3–4 years). The ADNC range in this group was from A1B1C0 in L‐AD to A3B3C3 in H‐AD. In the FTLD group, no apparent differences were seen between the time of the scan and death (1‐–4 years). The PSP‐1 case had an ADNC classification of A0B0C0 (no ADNC neuropathology), whereas the PSP‐2 case was A2B2C3 (low‐intermediate ADNC).

### 3.2 Immunohistochemical tau staining

**FIGURE 1 alz14330-fig-0001:**
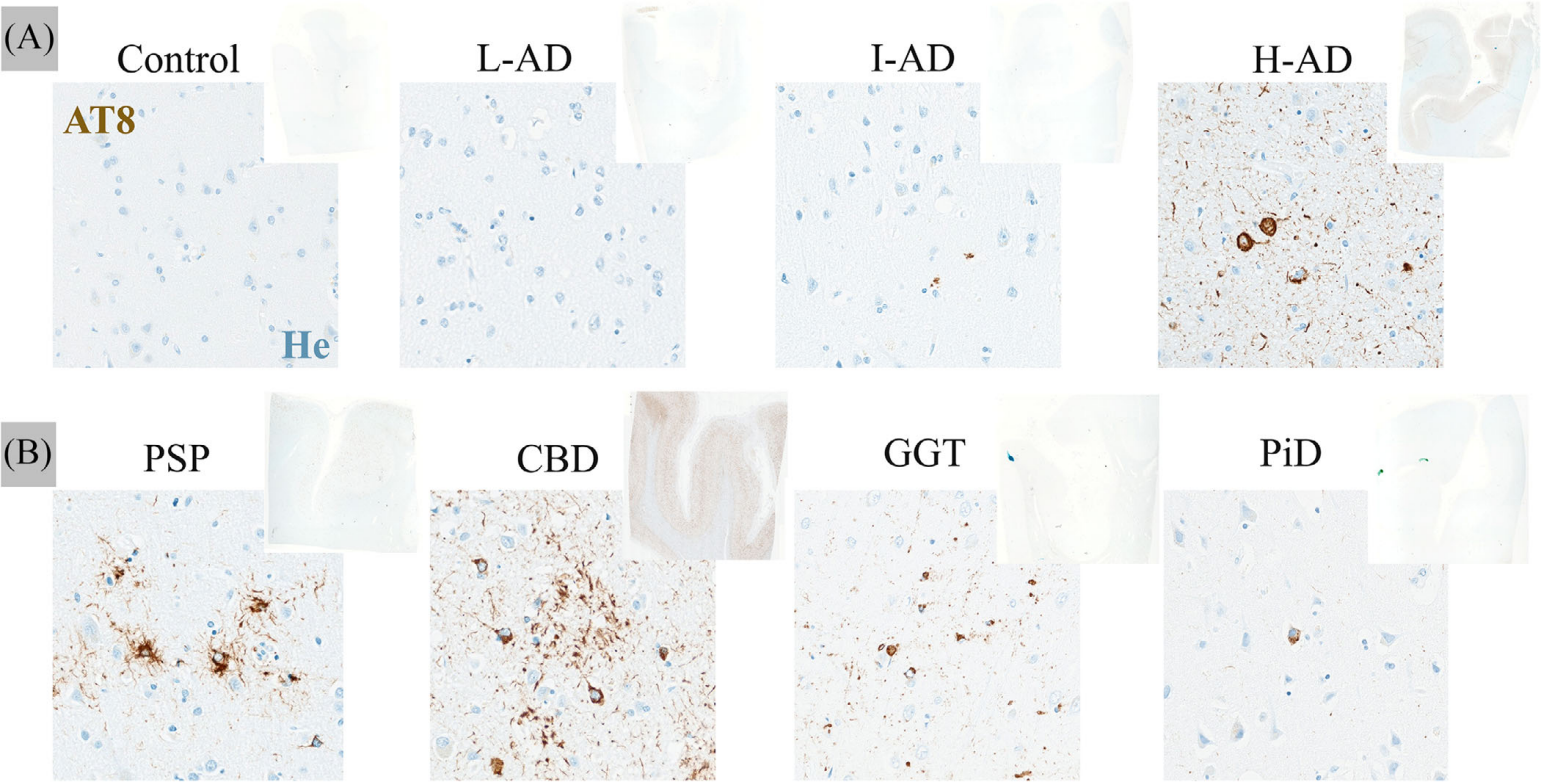
Immunohistochemical details of tau (AT8) staining and hematoxylin (He) from the SFMG region. (A) Control and AD cases and (B) FTLD cases. Note that topographic upper offset pictures are included. Scale bar = 10 µm. AD, Alzheimer's disease; CBD, corticobasal degeneration; FTLD, frontotemporal lobar degeneration; GGT, glial globular tauopathy; H‐AD, high‐likelihood AD; I‐AD, intermediate‐likelihood AD; L‐AD, low‐likelihood AD; PiD, Pick's disease; PSP, progressive supranuclear palsy; SFMG, superior frontal motor gyrus.

Staining with AT8 tau antibody showed variable amounts of tau immunoreactive inclusions in SMFG in eight cases (all but the control) (Figure 1). Across the three AD cases, the highest burden of tau immunoreactivity was found in the H‐AD case, followed by a progressive decrease in staining in the I‐AD case and then the L‐AD cases in the temporal and hippocampal regions. In the FTLD group, AT8 detected moderate tau‐positive lesions in both PSP cases, and the CBD and GGT cases, with less staining observed in the PiD case. In the hippocampus, the PSP‐2 case showed slightly increased staining across granular cells of the dental gyrus compared to the PSP‐1 case, which is unsurprising given that the PSP‐2 case had some ADNC. In this region, more significant staining was observed in the CBD case, which showed increased neurofibrillary threads. For the PiD case, in addition to neurofibrillary threads, we observed staining of many Pick's bodies, which were also present across the temporal cortex. We observed several astrocytic lesions in the PSP‐2 and CBD cases in the temporal cortex associated with neurofibrillary tangles (NFTs). Astrocytic and NFT lesions were also observed in the hippocampus and temporal cortex in the GGT case. In the BG, astrocytic lesions were observed in the PSP, CBD, and GGT cases. No lesions were found across the BG region of the PiD case (Figure S1).

### 3.3 T726 versus 6F/3D (Aβ) and Ab39 (AD specific tau) in H‐AD

**FIGURE 2 alz14330-fig-0002:**
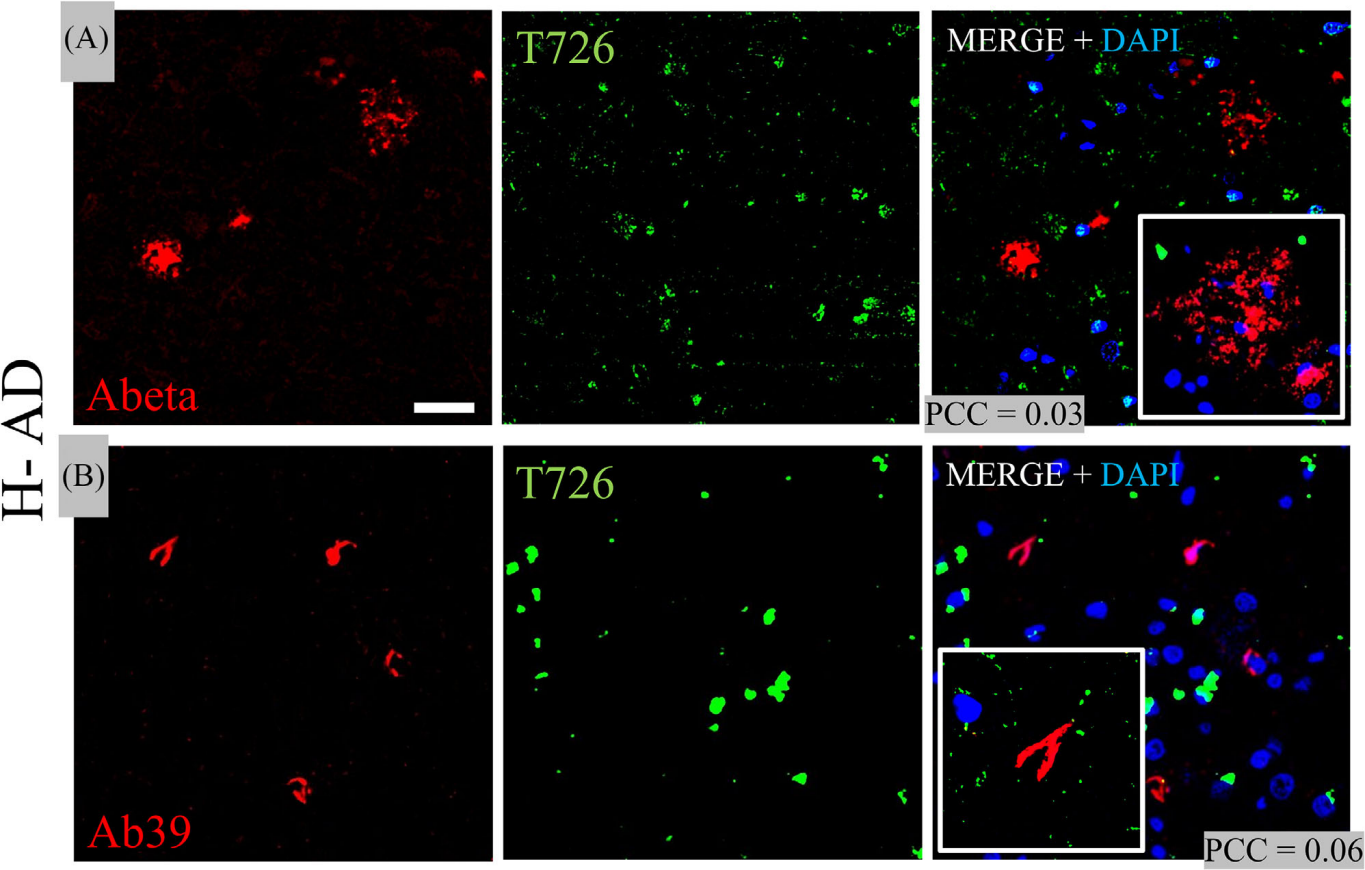
T726 staining suggests that tau‐PET is not binding to amyloid beta (Aβ) and the mature tau isoform in the temporal cortex from a patient with H‐AD. (A) T726 is represented in greenand Aβ in red. (B) Representative confocal image of T726 and mature tau isoform marked by Ab39 in red. Insets with a higher magnification are included to demonstrate further microstructural details. Note the minimal co‐localization between T726 and Aβ or Ab39. Abeta (Aβ), antibody to identify amyloid plaques; Ab39, antibody to identify ghost tangles; AD, Alzheimer's disease; DAPI, 4′,6‐diamidino‐2‐phenylindole nuclear counterstaining; H‐AD, high‐likelihood AD; PCC, Pearson's co‐localization coefficient; PET, positron emission tomography.

The fluorescent T726 compound (green) showed binding to many lesions in the temporal cortex of the H‐AD case. In the H‐AD case, 6F/3D fluorescence (red) demonstrated the presence of positive Aβ lesions, and Ab39 fluorescence (red) demonstrated the presence of AD‐specific conformation tau. Confocal microscopy did not show any overlap between T726 and Aβ or between T726 and Ab39 (average PCC = 0.03 for T726 vs 6F/3D, and PCC = 0.06 for T726 vs Ab39) (Figure 2).

### 3.4 T726 co‐localizes with PHF‐1 in the hippocampus and temporal cortex in AD

**FIGURE 3:**
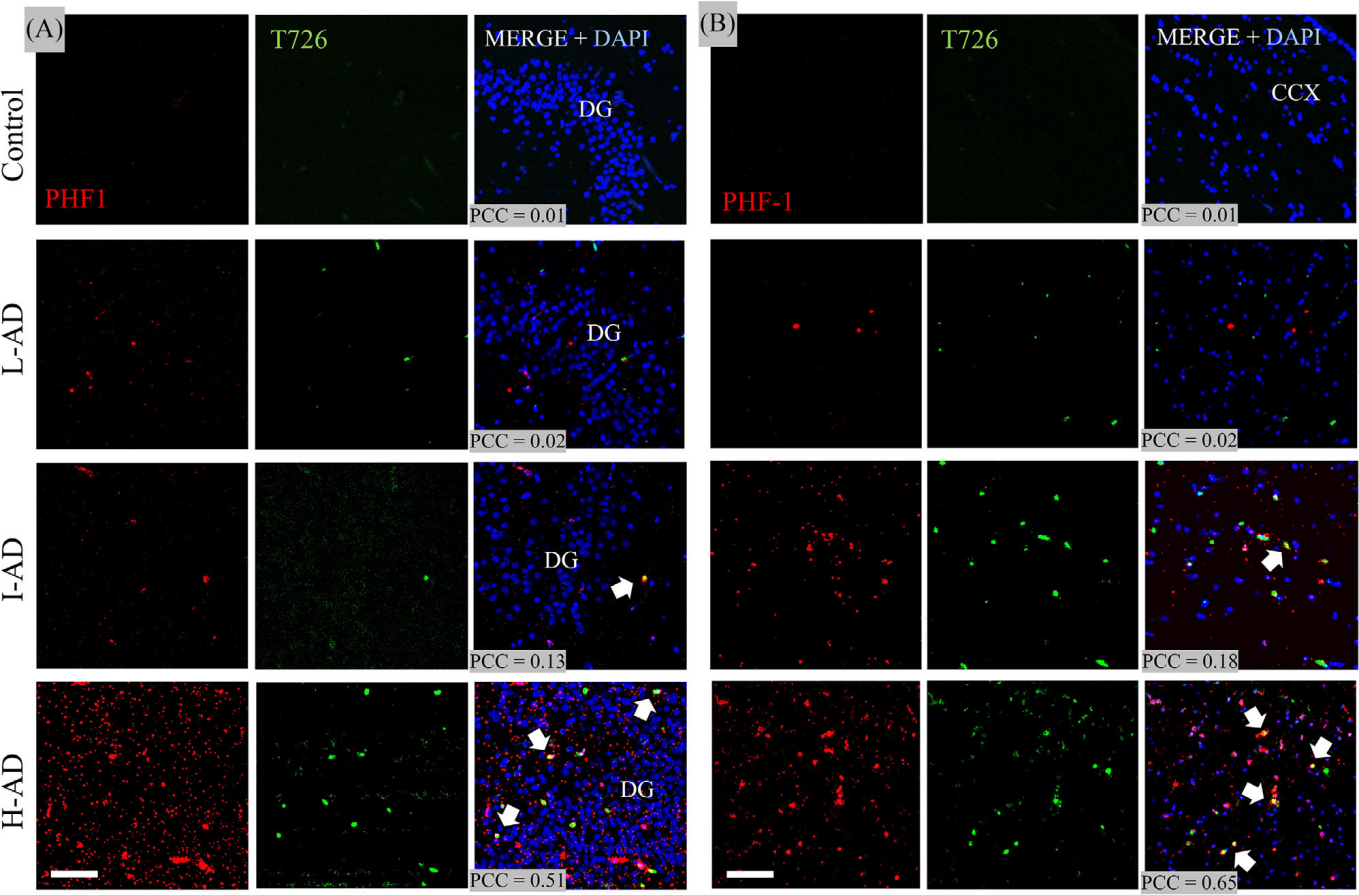
Confocal evaluation of T726 (green) and PHF‐1 (red) co‐localization across HC (A) and temporal cortical regions (B) from control; L‐AD; I‐AD; and H‐AD participants. Note that no significant PCC values were observed in the control regions, but progressive co‐localization between T726 and PHF‐1 across both study regions. AD, Alzheimer's Disease; CCX, temporal cortex; DAPI, 4′,6‐diamidino‐2‐phenylindole nuclear counterstaining; DG, dentate gyrus; H‐AD, high‐likelihood AD; HC, hippocampus; I‐AD, intermediate‐likelihood AD; L‐AD, low‐likelihood AD; PCC, Pearson's co‐localization coefficient; PHF‐1, paired helical filament 1; T726, fluorescent carbazole & FTP analog compound.

The fluorescent T726 compound (green) showed binding to lesions, increasing in burden as we went from L‐AD, I‐AD, and H‐AD in the hippocampus and temporal cortex (Figure 3). A similar pattern was seen with PHF‐1 (red). No lesions were observed in the control case with T726 or PHF‐1 in the hippocampus or temporal cortex. Confocal evaluation of T726 and PHF‐1 in control and three AD cases (L‐AD, I‐AD, and H‐AD) demonstrated co‐localization (or lack thereof) in the hippocampal region (left panel) with an average PCC of 0.01, 0.02, 0.13, and 0.51, respectively, as well as in the temporal cortex (right panel) again in increasing order from 0.01, 0.02, 0.18, and 0.65.

### 3.5 Evaluation of T726 versus PHF‐1 and 4R tau in FTLD cases

**FIGURE 4:**
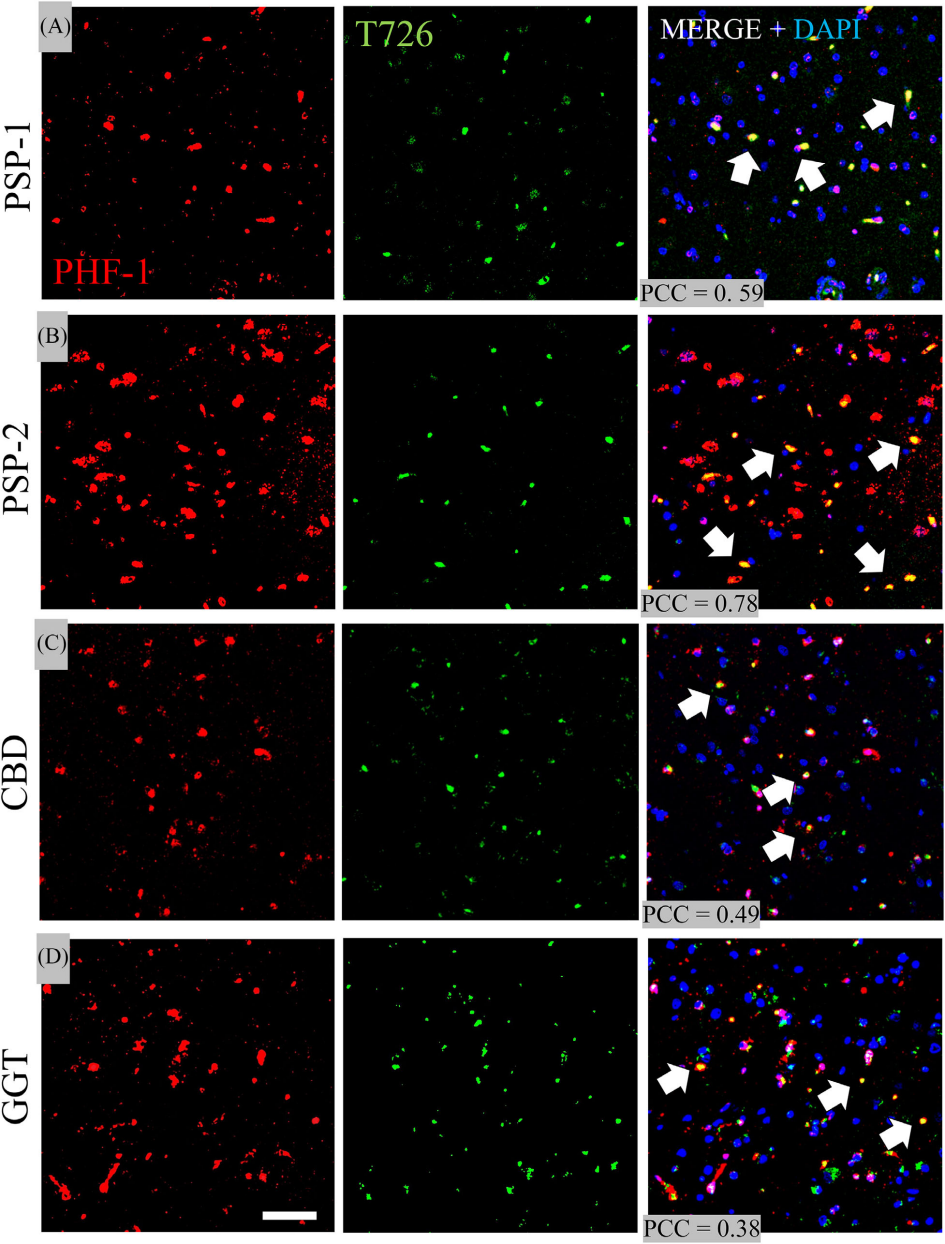
Evaluation of T726 and PHF‐1 binding. Representative confocal microscopy labeling of PHF1 (red) and T726 (green channel) across FTLD's SFMG ROIs. A, PSP‐1, B, PSP‐2; C, CBD, D, GGT subjects. Note that the largest colocalizations (white arrows) are among the PSP subjects (PCC values of PSP‐1 > PSP‐2) with lower PCCs from SFMG ROIs from the CBD and GGT cases. Scale bar = 10 µm. CBD, corticobasal degeneration; DAPI, 4′,6‐diamidino‐2‐phenylindole nuclear counterstaining; GGT, globular glial tauopathy; PCC, Pearson colocalization coefficient; PHF‐1, paired helical filament 1; PSP, progressive supranuclear palsy; ROI, region of interest; SFMG, superior frontal motor gyrus; T726, fluorescent carbazole & FTP analog compound.

The fluorescent T726 compound (green) showed binding to many lesions in all four 4R tauopathy cases (2 PSP, 1CBD, 1GGT) in the SFMG. PHF‐1 fluorescence (red) also detected lesions in the SMFG. Confocal microscopy labeling of PHF‐1 and T726 in the SFMG revealed co‐localization in both PSP cases, where PCC averaged 0.59 and 0.78, respectively. A lower overlap was seen in the CBD case, with an average PCC of 0.49, and a similar amount in GGT with an average PCC of 0.78 (Figure 4). In the temporal cortical region, PHF‐1 colocalization with T726 was larger in the PSP‐2 case. Overall, PHF‐1 co‐localization was consistently observed in the BG across cases. Meanwhile, fluorescence stainings with 4R tau showed considerable overlap in the temporal cortex of the PSP‐2 subject and the BG of the CBD case (Figure S2).

**FIGURE 5:**
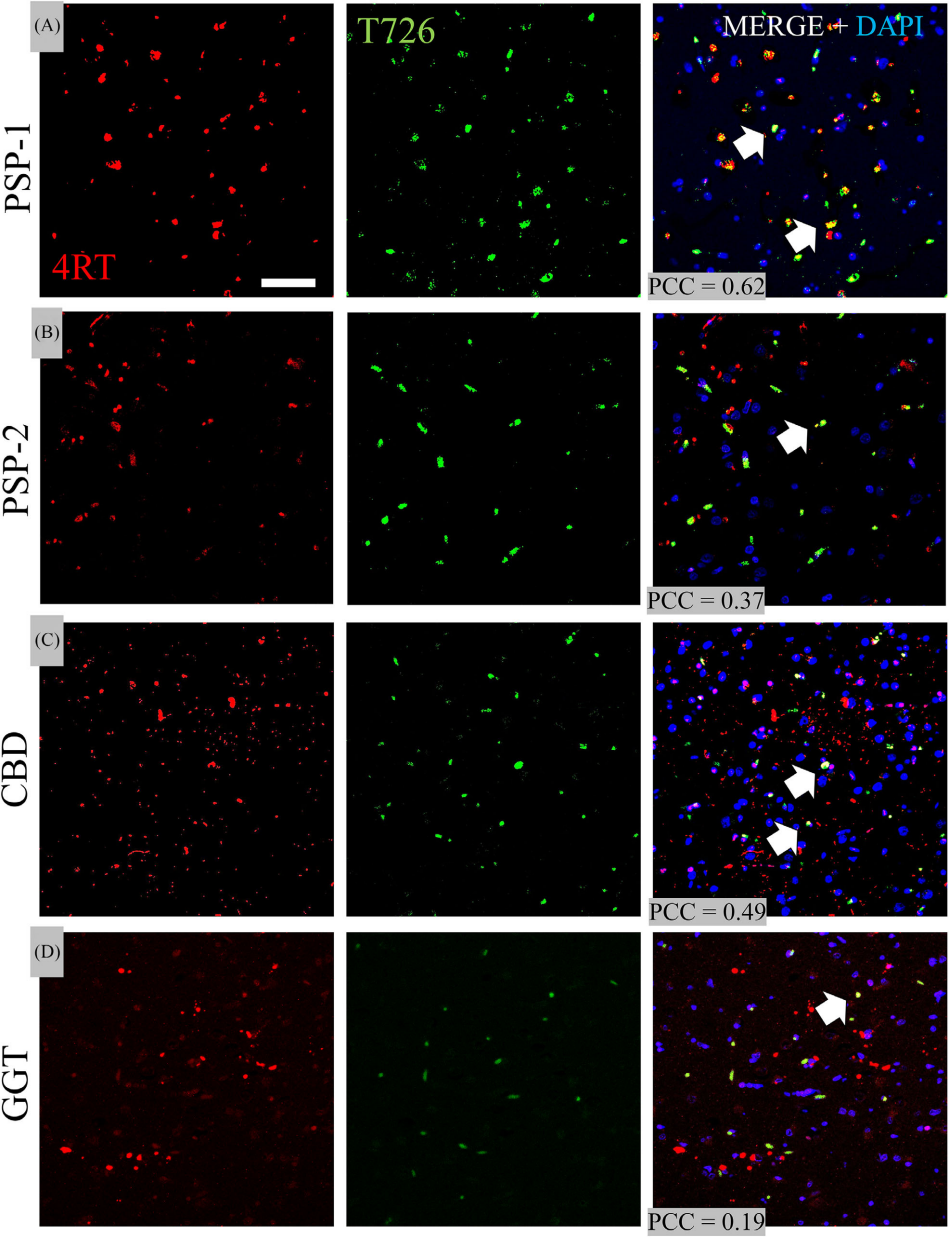
Evaluation of T726 and 4RT binding. Confocal microscopy details of 4RT stainings from comparable SFMG ROIs are shown in Figure 4. Different degrees of co‐localization among cortical neurons are pointed (white arrows). Note that the co‐localization ban can be seen between T726 and 4RT. Scale bar = 10 µm. 4RT, four repeat tau; CBD, corticobasal degeneration; DAPI, 4′,6‐diamidino‐2‐phenylindole nuclear counterstaining; GGT, globular glial tauopathy; PCC, Pearson colocalization coefficient; PSP, progressive supranuclear palsy; ROI, region of interest; SFMG, superior frontal motor gyrus; T726, fluorescent carbazole & FTP analog compound.

**FIGURE 6:**
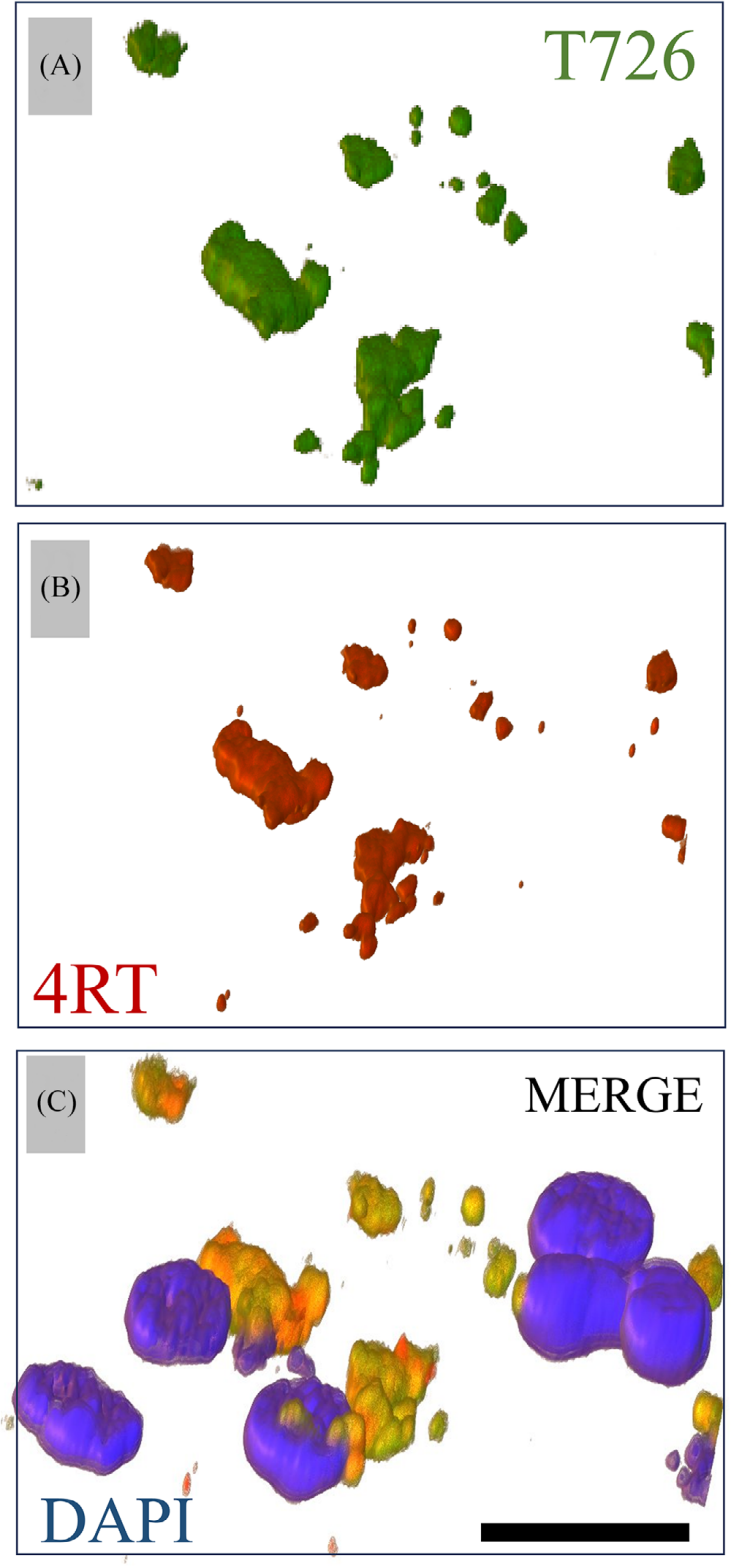
Confocal three‐dimensional (3D) rendering between FTP fluorescent analog and 4RT staining. Representative BG ROI from a patient with CBD showing 3D confocal z‐stack reconstruction of T726 (A) and 4RT (B) showing the overlap between the two channels (C). PCC = 0.89. Scale bar = 10 µm. 4RT, four repeat tau; BG, basal ganglia; CBD, corticobasal degeneration; FTP, ^18^F flortaucipir; ROI, region of interest.

In the evaluation of T726 (green) and 4RT (red), we observed some but a smaller amount of co‐localization compared to PHF‐1 in the two PSP and the CBD cases (average PCC = 0.62 and 0.37 in PSP and 0.49 in CBD, respectively). There was even less overlap in the GGT case (PCC = 0.19) (Figure 5). Additional 3D z‐stack confocal renderings from the BG of the CBD case show some overlap between T726 and 4RT (Figure 6). In the PiD case, we did not observe co‐localization between the T726 compound and PHF‐1 or T726 and 4RT in any of the brain regions assessed.

### 3.6 Evaluation of T726 versus 3R tau in PiD

**FIGURE 7:**
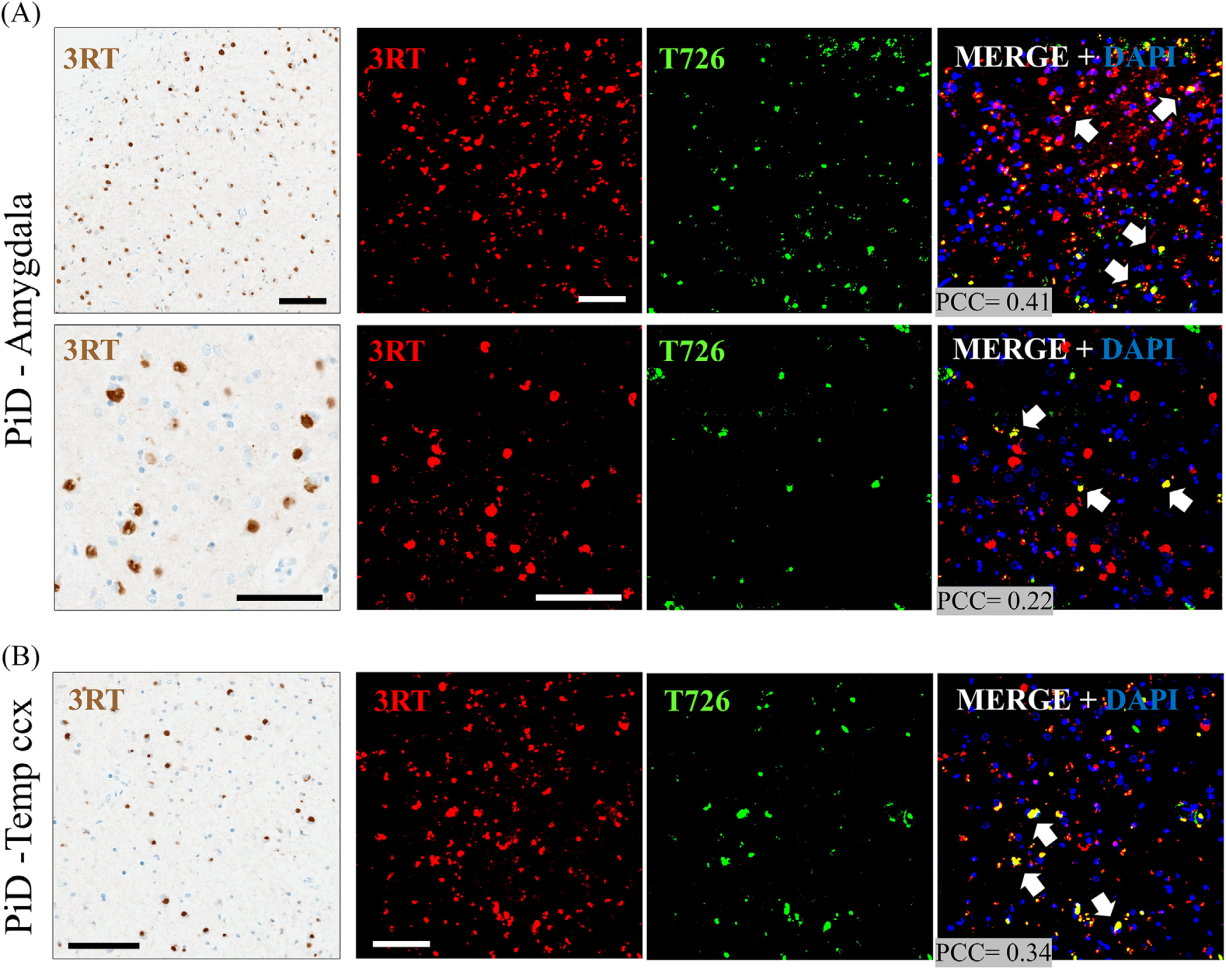
IHC staining from the amygdala and temporal cortex in the PiD case. (A) 3RT (right) DAB (3,3′‐diaminobenzidine) IHC staining from a PiD's representative amygdala region. Topographic (upper) and higher magnification (lower) areas showing positive Pick's bodies (scale bar = 100 microns). Additional fluorescence IHC with 3RT and T726 (left) demonstrated co‐localization between 3RT and T726 (scale bar = 20 microns). (B) Pick's bodies in the PiD subject from cortical temporal region (Temp‐ccx) showed similar co‐localization pattern between 3RT and T726 (white arrows). 3RT, three repeat tau; ccx, cortex; IHC, immunohistochemistry; PiD, Pick's disease.

Immunohistochemistry with 3,3′‐diaminobenzidine (DAB) i in the amygdala and lateral temporal regions in the PiD case showed positive Pick's bodies stained for 3RT. Fluorescence immunohistochemistry with the 3RT antibody showed a similar distribution pattern as observed with the DAB staining. Confocal analyses demonstrated co‐localization between 3RT and T726 in the amygdala (PCC = 0.41) and lateral temporal cortex (PCC = 0.22) areas, respectively (Figure 7).

## 4 DISCUSSION

This study's main finding is that T726 co‐localized with some 3R and 4R tau lesions in FTLD, suggesting that FTP is likely also binding to some 3R and 4R tau.

The use of FTP PET in current medical practice in AD is well established. FTP PET is being utilized as part of the clinical evaluation of patients with dementia. On the other hand, FTP uptake in FTLD has been a point of open debate. Questions such as whether it binds, how much binding occurs if it does bind, why it may bind to some 3R and 4RT lesions but not others, and whether there is region‐specific binding are unanswered. This makes FTLD more complicated because the distribution of staining in FTLD varies with clinical syndromes and likely varies with different underlying proteinopathy, co‐pathology, and pathology burdens. As such, clinically diagnosed patients suspected to have underlying FTLD may not have underlying 4RT or 3RT pathology, given that other proteins, TAR DNA‐binding protein 43 (TDP‐43) and FET, can also cause FTLD. Hence, it is almost impossible to address the questions above with autopsy tissue analysis. On the other hand, FTP is a ligand utilized in living individuals. Hence, to address these unanswered questions, we applied an analog of FTP to autopsy tissue.

Our experiments corroborate previous findings in the literature on the absence of binding of FTP to Aβ.^14^, ^15^ Of interest, although we found good overlap with lesions detected by PHF‐1, we did not find overlap with Ab39. PHF‐1 detects mature tau of all isoform types (3R+4R), whereas Ab39 detects AD (3R+4R) conformation‐specific lesions.^41^ It is still being determined why we did not see co‐localization between T726 and Ab39. One possible explanation is that Ab39 is binding Aβ and not tau. Another likely explanation is that Ab39 binds to a tau conformation in AD that T726 does not recognize. It is unlikely that Ab39 is binding to ghost tangles because FTP robustly detects ghost tangles.^15^, ^16^, ^47^ Considering our findings with PHF‐1 and assuming that Ab39 is binding a specific conformation of tau, it would appear that FTP uptake in AD does have a limitation in detecting specific conformations of PH‐t au in AD. Finally, another point to account for is the inter‐ and intra‐case differences reported previously in the study by Wren et al.^28^

The main aim of our study was to determine whether the evidence supports the notion that FTP does bind to some 3R and 4R tau lesions in FTLD‐tau. Before assessing this question, we show that T726 has the expected characteristics of FTP in AD. Regarding FTLD‐tau, we included cases of different 4RT or 3RT. We studied two patients with PSP, one with CBD and one with GGT, which are all 4RT variants, as well as PiD which is a 3RT. We did observe co‐localization of T726 with lesions stained with an antibody that recognizes 4RT lesions. This provides evidence supporting FTP binding to some 4RT lesions in FTLD. In the case of PiD, we did not observe co‐localization of T726 with 4R tau lesions (there were none). Additional co‐localization experiments with T726 and 3RT showed overlaps across temporal and amygdala regions, indicating the possibility that increased FTP signals seen in PiD cases could be due to FTP binding to 3R tau in PiD. With that said, we cannot exclude off‐target bindings.

Of interest, some but not all 3RT and 4RT lesions co‐localized with T726. This could explain the low uptake of FTP in FTLD tauopathies. The reason for the preferential binding to some but not all 3RT and 4RT lesions is unclear. FTP is like thioflavin‐S, which detects proteins rich in beta‐pleated sheets such as Aβ and NFTs in AD, and is similar to thioflavin‐T. Many neuropathologists use thioflavin‐S to detect, diagnose, and score ADNC. Thioflavin ‐T does preferentially detect proteins rich in beta‐pleated sheets.^48^ We, therefore, hypothesize that T726 and, hence, FTP could be detecting 3RT and 4RT aggregates that are rich in beta‐pleated sheets. Future studies should focus on understanding whether there are molecular and structural differences between the 3RT and 4RT lesions that are detected by T726 versus those that are not detected.

### 4.1 Strengths and limitations

This is one of the few studies exploring the potential binding of FTP to 3RT and 4RT lesions in FTLD‐tau. The limitations of this study include the limited number of samples available to make general statements and the potential nonspecificity binding among different antibodies that could have detected overlapping tau species. However, our optimization studies included the proper negative and positive controls, where each antibody was scrutinized at increasing concentrations. Moreover, although the application of our quencher successfully removed autofluorescence among our brain samples, it also partially deteriorated our fluorescent signal, which may have led to us underestimating co‐localization. It is also possible that our results are somewhat affected by off‐target binding to unknown factors associated with pathological tau. Other points to consider are differences in AT8 and PHF‐1 antibody binding to phosphorylated tau,^49^ which may add variability, and structural differences between T726 and FTP, which may affect our confocal results. Studies have reported chemical differences between FTP (7‐(6‐fluoropyridin‐3‐yl)‐5H‐pyrido[4,3‐b] indole) and T726 (*N*‐(2‐fluoroethyl)‐*N*‐methyl‐5H‐pyrido[3,2‐b] indol‐7‐amine) that affect differences in histological binding and hence the results of our experiments.^6^, ^28^ However, combined immunohistochemistry and in vitro autoradiography studies of human AD brain samples have shown that both FTP and T726 have higher binding to PHF‐tau aggregates.^50^

## 5 CONCLUSIONS

Our findings point toward FTP binding to some 3R and 4R tau species in FTLD. This may explain the observed increased uptake of FTP signals observed in some FTLD cases. Further studies are needed to determine why some 3R and 4R tau species but not others are recognized by FTP.

## AUTHOR CONTRIBUTIONS

Rodolfo G. Gatto and Keith A. Josephs contributed to the study's conception and design. Rodolfo G. Gatto and Youssef Hossam performed the fluorescent stainings. All authors contributed to the interpretation of the results. Rodolfo G. Gatto drafted the first version of the manuscript. All authors critically revised it for important intellectual content. All authors contributed significantly to the research and development of the manuscript and approved the final version for publication.

## CONFLICT OF INTEREST STATEMENT

The authors declare no competing interests. Author disclosures are available in the supporting information.

## CONSENT STATEMENT

All human subjects provided written informed consent.

## Supporting information



Supporting Information

Supporting Information

Supporting information
